# Progenitor cells from brown adipose tissue undergo neurogenic differentiation

**DOI:** 10.1038/s41598-022-09382-8

**Published:** 2022-04-04

**Authors:** Medet Jumabay, Li Zhang, Jiayi Yao, Kristina I. Boström

**Affiliations:** 1 Division of Cardiology, David Geffen School of Medicine at UCLA, Box 951679, Los Angeles, CA 90095-1679 USA; 2 Molecular Biology Institute, UCLA, Los Angeles, USA; 3 Division of Rheumatology, Allergy & Immunology, UCSD School of Medicine, La Jolla, CA 92093 USA

**Keywords:** Mesenchymal stem cells, Stem-cell differentiation

## Abstract

Multipotent cells derived from white adipose tissue have been shown to differentiate into multiple lineages including neurogenic lineages. However, the high innervation of brown adipose tissue by the sympathetic nervous system suggest it might be a better source of neural precursor cells. To investigate potential differences between white and brown progenitors, we cultured white and brown dedifferentiated fat (wDFAT and brDFAT) cells from mouse and human adipose tissue and compared marker expression of neural precursors, and neuronal and glial cells, using fluorescence-activated cell sorting, bright-field imaging, immunofluorescence, and RNA analysis by qPCR. The results showed that both wDFAT and brDFAT cells had the capacity to generate neuronal and glial-like cells under neurogenic conditions. However, the brDFAT cells exhibited enhanced propensity for neurogenic differentiation. The neurogenic cells were at least in part derived from Adiponectin-expressing cells. TdTomato-expressing cells derived from *Adiponectin (Adipoq) Cre* ^ERT2^ -*tdTomato* ^flox/flox^ mice gave rise to individual cells and cell clusters with neurogenic characteristics. Moreover, human brDFAT cells demonstrated a similar ability to undergo neurogenic differentiation after treatment with neurogenic medium, as assessed by immunofluorescence and qPCR. Together, our results support that brDFAT cells have ability to undergo neurogenic differentiation.

## Introduction

Adipose tissue-derived cells provide a source of different stem cells for tissue regeneration and repair, including neural precursor cells. Adipose stromal cells derived from white adipose tissue (WAT) have been shown to generate early neural progenitor cells that are able to differentiate into oligodendrocytes, astrocytes and neurons^1^. Furthermore, dedifferentiated fat (DFAT) cells from WAT were shown to increase functionality after spinal cord injury in mice^2^. Although these studies showed potential for neurogenic differentiation in cells from WAT, no studies have examined whether a similar potential exists in cells derived from brown adipose tissue (BAT) despite its high degree of innervation.

Adipose tissue is traditionally classified as white or brown, with distinct cell lineages of white or brown adipocytes^3,4^. White adipocytes are characterized by a large single, spherical vacuole with few mitochondria. The easy access to WAT has made it a target tissue for the generation of ASCs and DFAT cells. Brown adipocytes, on the other hand, are characterized by multiple, multilocular lipid droplets, and the ability of thermogenic activity and high density of mitochondria. These are instrumental in dissipating stored energy as heat through activation of the uncoupling protein-1 (UCP-1) that “uncouples” fuel oxidation from ATP synthesis. BAT is highly vascularized and sympathetically innervated as the sympathetic nervous system is instrumental in regulating the thermogenic process^5–8^. BAT also varies between species and age groups. In humans, BAT was first identified in infants and thought to be lacking in adults. However, later data showed significant depots of genuine BAT in the supraclavicular and spinal regions of human adults^9^.

The distinction between brown and white adipose tissue suggest there may be differences in the propensity for specific cell lineages in progenitor cells derived from BAT and WAT. The high level of sympathetic innervation and vascularization may point to BAT being a better source of neural precursor cells. Here, we examine whether BAT-derived progenitor cells from mouse and human are able to undergo neurogenic differentiation, as compared to progenitor cells derived from WAT.

## Material and methods

### Mouse and human adipose tissue

To generate mice with expression of tandem dimer (td)Tomato red fluorescent protein directed by the *Adiponectin* (*Adipoq*) promoter, we crossed C57BL/6-Tg(Adipoq-cre/ERT2)1Soff/J mice (Jackson Laboratory, stock number 025124) and B6;129S6-*Gt(ROSA)26Sor*^*tm*9* (CAG-tdTomato)Hze*^/J mice (Jackson Laboratory, stock number 007905) to generate Tg(Adipoq-cre/ERT2) ; Gt(ROSA)26Sor^tm9 (CAG-tdTomato)Hze^/J mice (referred to as *Adipoq*-cre/ERT2;*tdTomato*) mice. The mice were treated with Tamoxifen for 7 days as previous described^10^, starting at 7–8 weeks of age. Adipose tissue for cell isolation was collected from wild type C57BL/6 J mice or *Adipoq*-cre/ERT2;*tdTomato* mice at 10–12 weeks of age after euthanasia by inhalation of isoflurane (5–30%) followed by cervical dislocation. The studies were reviewed and approved by the Institutional Review Board and conducted in accordance with the animal care guidelines set by the University of California, Los Angeles (UCLA). The study was carried out in compliance with the ARRIVE guidelines. The investigation conformed to the National Research Council, *Guide for the Care and Use of Laboratory Animals, Eighth Edition* (Washington, DC: The National Academies Press, 2011). De-identified autopsy samples from human infants (less than 3 months) with interscapular BAT and subcutaneous WAT were used for cell preparation. Since the research was using de-identified autopsy samples, the UCLA Office of the Human Research Protection determined that it did not meet the definition of human subjects research and did not require review.

### Cell preparation from brown and white adipose tissue

For mice, WAT was collected from the anterior subcutaneous and inguinal fat depots of the mice^11^, and BAT was collected from the interscapular region. Three mice were used for each cell preparation. In order to prevent contamination, each tissue type was collected using separate autoclaved instruments and placed in their respective 50 mL tubes containing phosphate-buffered saline (PBS). Afterwards, WAT and BAT were separately washed three time in PBS, until clear, and then minced in 10 cm Petri dishes with 15 mL of 0.2% collagenase, 2% bovine serum albumin (BSA) in Dulbecco’s modified Eagle’s medium (DMEM) (10–013-CV, Corning Life Sciences). Human infant autopsy specimens were similarly processed. A sample from each specimen was examined by H&E staining and UCP1 expression prior to the use of the cells to ensure the expected morphology and UCP1 expression.

White and brown minced adipose tissue in collagenase were then transferred to new 50 mL test tubes where they were further digested while agitated (85 rpm) for 45 min at 37 °C. Next, the cell suspensions were filtered into 10 cm Petri dishes and washed twice with 10 mL of culture medium [DMEM containing 20% fetal bovine serum (FBS) and 0.5% penicillin–streptomycin]. Each suspension was then poured into a new 50 mL test tube. In order to isolate multipotent cells from the white and brown adipocyte fractions referred to as dedifferentiated fat (DFAT) cells^12,13^, the test tubes containing each respective adipose tissue were centrifuged for 3.15 min at 12,000 rpm. After centrifugation, the top cellular layer containing adipocytes was collected and added to a new 50 mL test tube with 10 mL of culture medium. The test tubes were centrifuged for 1 min. Adipocytes (30–50 µl of the top cellular layer) were then added to culture medium in 6-well plates fitted with 70 µm-filters and incubated for 5 days. DFAT cells generated from the adipocytes passed through the filters and attached to the bottom of the dishes. After 5 days, the filters with remains of the adipocytes were removed. This method allowed the separation of the DFAT cells from the adipocytes as soon as they passed through the filter and attached to the bottom of the dish where they grew to confluence. The cells were referred to as white (w)DFAT or brown (br)DFAT cells. The cells were subsequently plated at 75% confluency for experiments. Where indicated, the cells were cultured in 3D-Matrigel Growth Factors Reduced (GFR) basement membrane matrix (Corning Inc.).

### Neural induction

After reaching 100% confluence, brDFAT and wDFAT cells were passaged onto 12-well dishes or 8-well glass chamber slides that were coated with 0.1% gelatin at a density of 2.5 × 10^4^ cells/cm^2^. The cells were plated in pre-inducing medium [DMEM supplemented with 20% KnockOut™ Serum Replacement (10,828,028; Gibco™) and 0.5% penicillin–streptomycin] for 7 days. On Day 7, the cells were washed twice with PBS and the medium was changed to basic control medium or neural induction medium. The cells plated in basic control medium [DMEM with 10% FBS and 0.5% penicillin–streptomycin] were maintained in this medium for up to 15 days with medium changes every 3 days. The cells plated in neural induction medium [1:1 ratio of DMEM and Ham’s F12 Nutrient Mixture (F12) (11,765,070; Gibco™) with 1% non-essential amino acids, 2% B-27 Supplement (17,504–044; Gibco™), 1% N-2 Supplement, 0.1 mM 2-mercaptoethanol, and 20 ng/mL recombinant mouse fibroblast growth factor basic (bFGF; 3139-FB; R&D Systems)], were maintained in this medium for up to 15 days with medium changes every 3 days.

### Flow cytometric analysis

For phenotypic characterization of the brDFAT and wDFAT cells, the cells were cultured in culture medium until 100% confluent. Fluorescence-activated cell sorting (FACS) analysis was performed after the first passage as previously described^14^ using the following conjugated anti-mouse antibodies: Sca1 (Ly-6A/E) [Phycoerythrin (PE), BD Biosciences, Cat. No. 553336), c-Kit (CD117) [Fluorescein isothiocyanate (FITC), BD Biosciences, Cat. No. 553354], CD105 (PE/Cyanine7, BioLegend, Cat. No. 120409), CD11b (Alexa Fluor 488, BioLegend, Cat. No. 101219), CD90 (PE, eBioscience, Cat. No. 12–0900-81), SSEA-1 (CD15) (Alexa Fluor 488, BioLegend, Cat. No. 125609), CD31 (PECAM1) (PE, BD Biosciences, Cat. No. 553373) and CD34 (FITC, eBioscience, Cat. No. 11–0341-85), all diluted 1:100 in 1% BSA.

### RNA analysis

RNA was extracted when (i) the cells were 70% confluent in the culture medium, (ii) after 7 days of culture in the pre-inducing medium, or (iii) after 3, 6, or 12 days in the basic medium or (iv) the neural induction medium. Real-time polymerase chain reaction (qPCR) was performed as previously described^14,15^. Primers and probes for RNA transcripts were supplied by Life Technologies as part of *TaqMan* Gene Expression Assays. The mouse primers and probes included Tubulin Beta 3 Class III (βTubIII), Nestin, Neurogenic Differentiation 1 (NeuroD1), S100 Calcium Binding Protein B (S100B), Glial Fibrillary Acidic Protein (GFAP), Oligodendrocyte Transcription Factor 2 (OLIG2), Uncoupling Protein 1(UCP1), and Glyceraldehyde 2-Phosphate Dehydrogenase (GAPDH). The human primers and probes included NeuroD, Neurofilament (NFL) and UCP1.

### Immunofluorescence

Immunofluorescence was performed on brDFAT and wDFAT cells that had been cultured in 24-well dishes or 8-well glass chamber slides coated with 0.1% gelatin. The cells were washed three times in PBS and fixed in 4% paraformaldehyde overnight at 4 °C. Mouse adipose tissue and cell clusters from 3D-Matrigel cultures were fixed, embedded in paraffin, and sectioned, before immunofluorescence. The next day, the cells were washed three times with PBS and permeabilized with 0.2% Triton™ X-100 for 20 min. The cells were washed once and blocked for 1 h with 10% chicken or goat serum in 1% BSA depending on the host of the secondary antibodies. Afterwards, the blocking buffer was removed and the cells were incubated overnight at 4 °C with the appropriate antibodies.

The following primary antibodies were used (diluted in 1% BSA in PBS): mouse anti-Neuron-specific βIII Tubulin (Clone #TuJ-1; R&D Systems, Cat. No. MAB1195, dilution 1:100), rabbit anti-S100B (Abcam, Cat. No. ab52642, dilution 1:100), rabbit anti-GFAP (Millipore Sigma, Cat. No. G9269, dilution 1:1,000), rabbit anti-Nestin (Santa Cruz Biotechnology, Cat. No. sc-20978 (H-85), dilution 1:100), mouse UCP1 (R&D Systems, Cat. No. MAB6158, dilution 1:200), rabbit anti-Perilipin (Cell Signaling, Cat. No. 9349, dilution 1:200), rabbit anti-Troponin I (Santa Cruz Biotechnology, Cat. No. sc-15368 (H-170), dilution 1:200), goat anti-NeuroD (R&D Systems, Cat. No. AF2746, dilution 1:200), and mouse anti-Neurofilament (NFL) (Thermo Fisher Scientific, Cat. No. 13–0400, dilution 1:200).

The next day, cells were washed repeatedly with PBS and incubated with the appropriate secondary antibody and DAPI for 1 h. Alexa Fluor (AF) 594-conjugated (red fluorescence) chicken anti-mouse (1:1000, Invitrogen/ThermoFisher, Cat. No. A-21201), AF 488-conjugated (green fluorescence) chicken anti-rabbit (1:1000, Invitrogen/ThermoFisher, Cat. No. A-21441) in 1% BSA, or AF 594 or AF 488-conjugated goat anti-mouse (1:1000, Invitrogen/ThermoFisher, Cat. No. A32742 or A32723), AF 488-conjugated goat anti-hamster (1:1000, Invitrogen/ThermoFisher, Cat. No. A-21110), AF 594 or AF 488-conjugated goat anti-rabbit (1:1000, Invitrogen/ThermoFisher, Cat. No A32740 or A-11008) in 1% BSA. The cells were washed with PBS and the nuclei were stained with 4’,6- diamidino-2-phenylindole (DAPI, Sigma-Aldrich, Cat. No. D9542). Afterwards, the cells were again washed with PBS and then imaged.

### Statistical analysis

Data were analyzed for statistical significance by unpaired t-tests or two-way ANOVA using GraphPad Prism 6.0 software (GraphPad Software, San Diego, CA, https://www.graphpad.com/scientific-software/prism). *P-*values less than 0.05 were considered significant.

## Results

### Detection of neurogenic markers in adipose tissue

**Figure 1:**
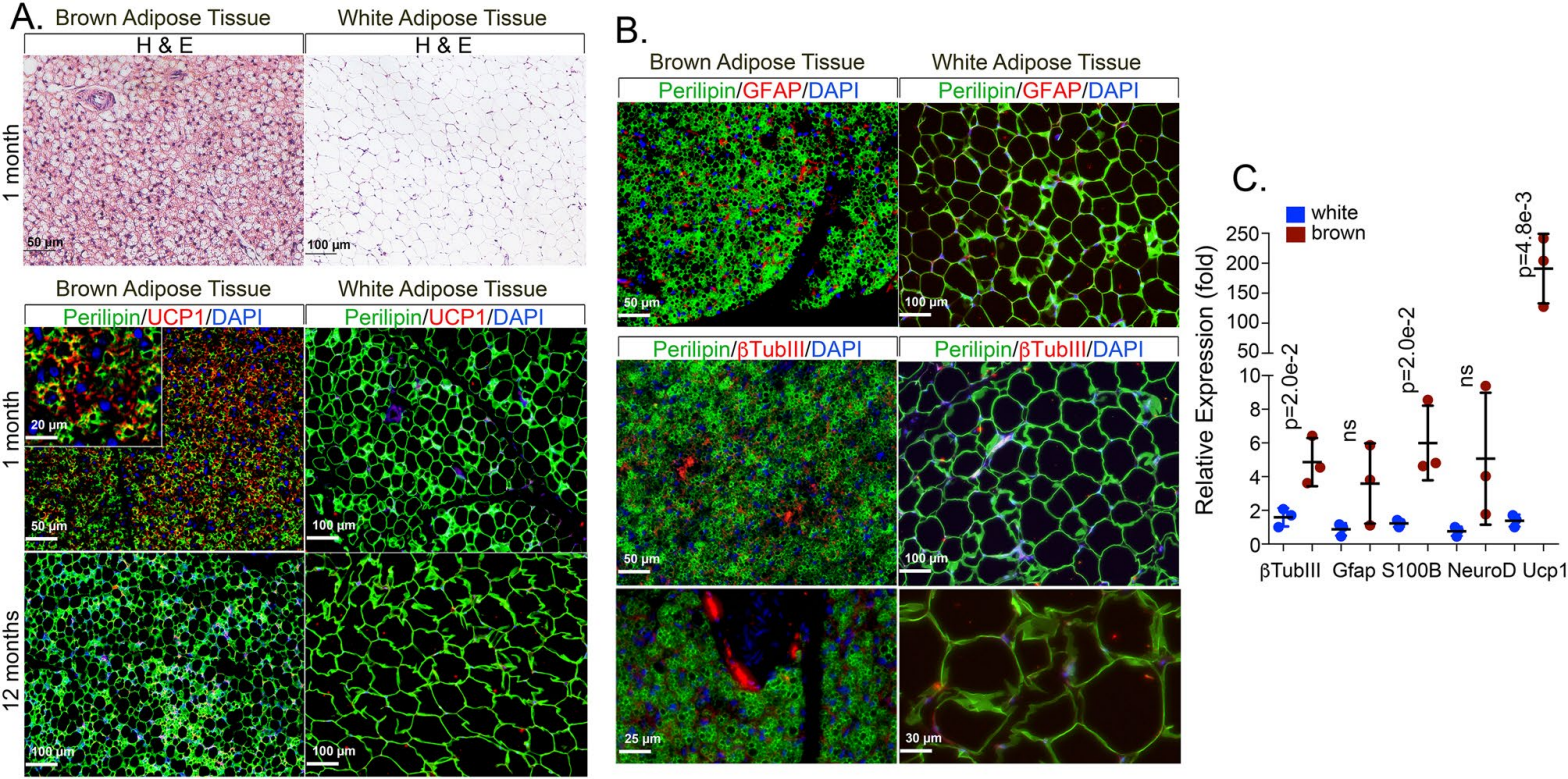
Enhanced expression of neurogenic markers in mouse brown adipose tissue. (**A**) H&E staining of mouse BAT (interscapular) and WAT (inguinal) (top). Expression of perilipin (green) and uncoupling protein 1 (UCP1, red) in brown and white adipose tissue from mice aged one month and 12 months as detected by immunofluorescence. (**B**) Expression of perilipin (green) and the neurogenic markers glial fibrillary acidic protein (GFAP, red) and β tubulin III (βTubIII, red) in brown and white adipose tissue from mice aged one month, as detected by immunofluorescence. (**C**) Expression of βTubIII, GFAP, S100 Calcium Binding Protein B (S100β), Neurogenic Differentiation 1 (NeuroD1), and UCP1 in brown and white adipose tissue from mice aged one month, as determined by qPCR (n = 3 tissue samples). DAPI (blue) was used for visualization of nuclei. *P*-values for statistically significant differences are indicated (unpaired t-tests); ns, non-significant.

BAT is highly innervated by the sympathetic nervous system as compared to WAT, suggesting that it might be a better source of neural precursor cells. As expected, the BAT showed higher UCP1 expression than WAT at one month of age, as determined by immunofluorescence and qPCR (Fig. 1A, C), but the UCP1 expression was less prominent at 12 months of age by immunofluorescence (Fig. 1A). At one month of age, the BAT also stained for the neurogenic markers GFAP and βTubIII (Fig. 1B). The expression of βTubIII, GFAP, NeuroD and the astrocyte marker S100β was significantly higher in BAT by qPCR, as compared to WAT (Fig. 1C).

### Expression of cell surface markers in mouse brDFAT and wDFAT cells

**Figure 2:**
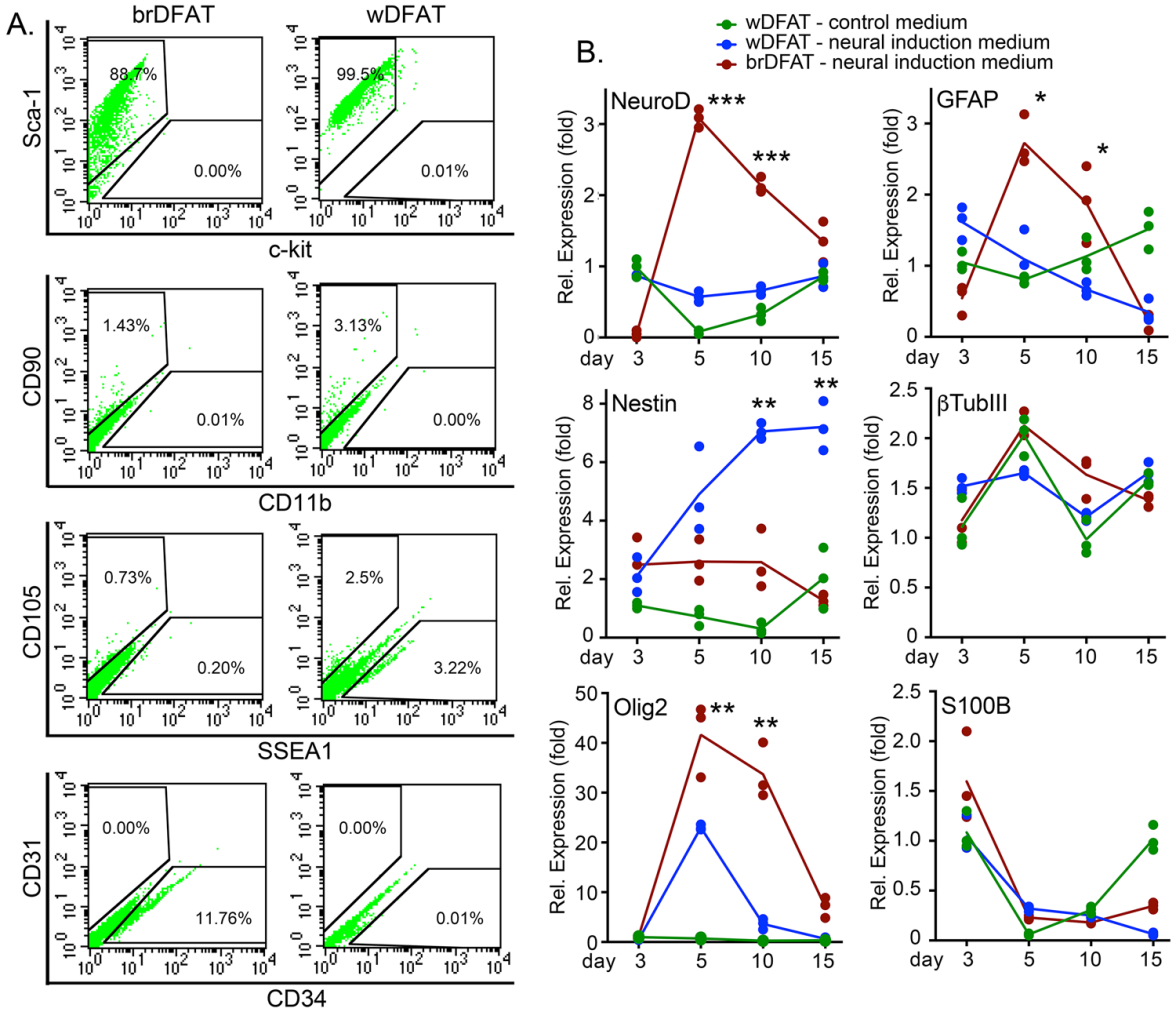
Expression of precursor cell markers in mouse brDFAT and wDFAT cells. (**A**) Both brDFAT and wDFAT cells showed high expression of Sca-1 as determined by FACS after one week of culture. Low expression of CD90, CD105, and SSEA1 was found in both cell types. However, only the brDFAT cells expressed the endothelial progenitor marker CD34. Representative of 3 experiments. (**B**) Time course expression of the neurogenic markers Neurogenic Differentiation 1 (NeuroD1), glial fibrillary acidic protein (GFAP), Nestin, β tubulin III (βTubIII), Oligodendrocyte Transcription Factor 2 (OLIG2), and S100 Calcium Binding Protein B (S100β) in brDFAT and wDFAT cells, as determined by qPCR. The cells were treated with neural induction medium; wDFAT cells in basic control medium were used for comparison (day 3 was set to 1) (n = 3 cell preparations. *P*-values for statistically significant differences are indicated (two-way ANOVA). * < 0.05, ** < 0.01, *** < 0.001.

We prepared brDFAT and wDFAT cells and briefly characterized their surface markers by FACS after one week of culture. The multipotency marker Sca1 was abundantly expressed in both brDFAT and wDFAT cells, 88.7% and 99.5%, respectively (Fig. 2A), consistent with previous results for wDFAT cells^16^. However, the multipotency marker SSEA1 was only expressed in 0.20% and 3.22% of brDFAT and wDFAT cells, respectively (Fig. 2A), and cKit, another marker of multipotency, was not expressed in any of the cell types. CD90, a partial determinant for undergoing neurogenic differentiation, was expressed in 1.43% and 3.13% of brDFAT and wDFAT cells, respectively. In addition, 11.76% of the brDFAT cells expressed the endothelial progenitor marker CD34 but none of the cells expressed the endothelial marker CD31 (Fig. 2A). CD105 (Endoglin) was expressed in 0.73% and 2.5% of the brDFAT and wDFAT cells, respectively (Fig. 2A). The macrophage marker CD11b was undetectable. Thus, the surface marker expression showed relatively small differences between the two cell types, although the expression of the endothelial progenitor marker was higher in the brDFAT cells, consistent with the higher vascularization of BAT.

### Induction of neurogenic differentiation

**Figure 3:**
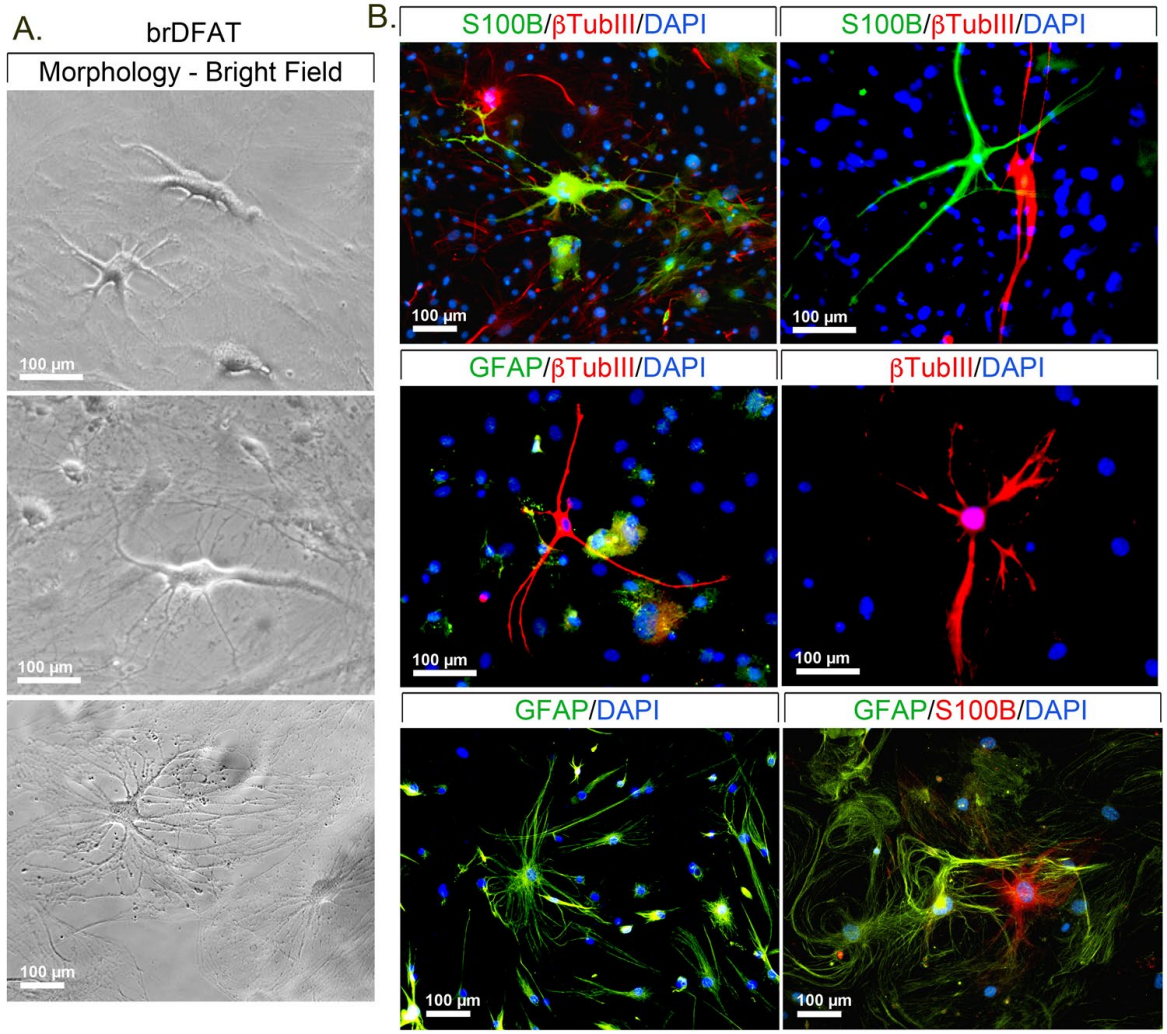
Mouse brDFAT cells in culture with neurogenic characteristics. (**A**) Bright field images of brDFAT cells after 15 days of culture in neural induction medium. (**B**) Expression of S100 Calcium Binding Protein B (S100β, green), β tubulin III (βTubIII, red), and glial fibrillary acidic protein (GFAP, green) as visualized by immunofluorescence in brDFAT cells after 15 days of culture in neural induction medium. DAPI (blue) was used to visualize nuclei. Representative images from 3–5 experiments.

To determine if the mouse brDFAT and wDFAT cells were capable of undergoing neurogenic differentiation, the cells were plated at approximately 75% confluency and first treated with pre-induction medium for 7 days, followed by neural induction medium for up to 15 days. WDFAT cells in basic control medium was used for comparison. The results showed significant induction of the neurogenic markers NeuroD, GFAP, Nestin and Olig2 on day 5–10 in the brDFAT cells, as compared to wDFAT cells in basic control or neural induction medium (Fig. 2B). There was no significant difference in the expression of βTubIII or S100β between the different cells (Fig. 2B). The results suggest that the brDFAT cells have a higher propensity for neurogenic differentiation. We confirmed the neural morphology of the brDFAT cells using immunofluorescence and antibodies against βTubIII, S100β and GFAP. BrDFAT cells with neural-like morphology appeared after 10–15 days of culture in the neural induction medium, as shown by bright field microscopy (Fig. 3A). Immunofluorescence confirmed expression of βTubIII, S100β and GFAP as shown in Fig. 3B. The staining for βTubIII, S100β and GFAP was found in separate cells, suggesting the differentiation was not uniform. The neuron-like cell population was less than 5% of the total cell population as determined by counting the βTubIII-positive cells. Thus, our results support that a subpopulation of the brDFAT cells is able to undergo neurogenic differentiation.

### Lineage tracing of neuron-like cells from Adiponectin-expressing cells

**Figure 4:**
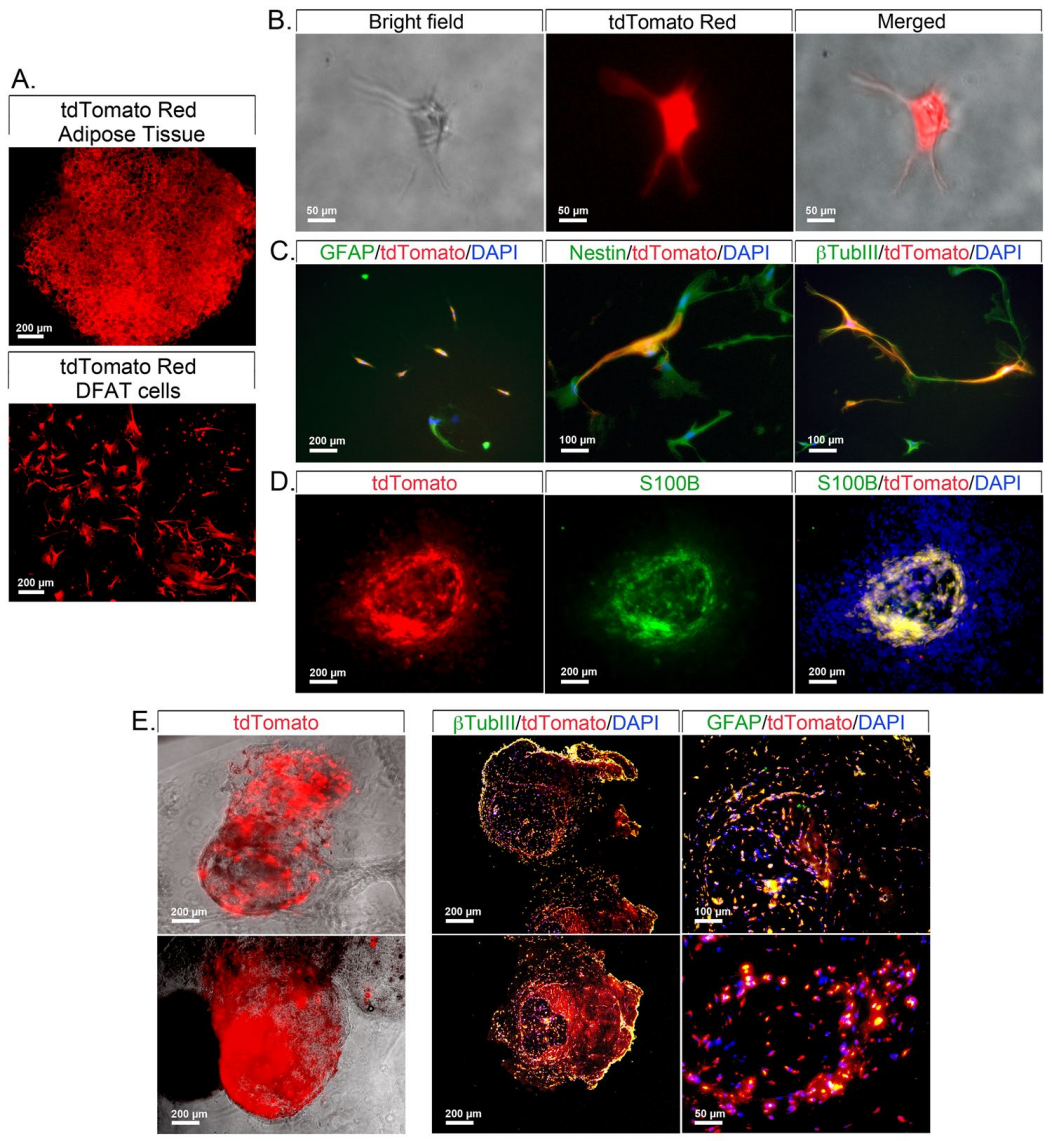
Mouse DFAT cells derived from combined BAT and WAT from Adipoq-cre/ERT;tdTomato mice after Tamoxifen induction. (**A**) TdTomato subcutaneous adipose tissue and mixed DFAT cells from *Adipoq*-cre/ERT2;*tdTomato* mice. (**B**) Bright field images of tdTomato DFAT cells after 15 days of culture in neural induction medium. (**C**) Co-localization of tdTomato (red) and the neurogenic markers glial fibrillary acidic protein (GFAP, green), Nestin (green), and β tubulin III (βTubIII, green) in DFAT cells after 15 days of culture in neural induction medium as visualized by immunofluorescence. (**D**) Co-localization of tdTomato and S100 Calcium Binding Protein B (S100β, green) in aggregates of tdTomato-positive DFAT cells as visualized by immunofluorescence. (**E**) Staining for βTubIII and GFAP in sections from DFAT cell clusters formed in 3D-Matrigel. DAPI (blue) was used to visualize nuclei. Representative images from 3–5 experiments.

To determine if the neuron-like cells derived from Adiponectin-expressing cells, we obtained subcutaneous adipose tissue (WAT and BAT) from the *Adipoq*-cre/ERT2;*tdTomato* mice after Tamoxifen-mediated tdTomato induction. We prepared DFAT cells from combined white and brown adipose tissue (see Fig. 4A for tdTomato-positive adipose tissue and cells), and placed them in regular culture or in 3D-Matrigel with neural induction medium. In regular cultures, cells with neuron-like morphology that expressed tdTomato emerged after 14 days (Fig. 4B.) Immunofluorescence revealed that tdTomato was co-expressed with the neurogenic markers GFAP, Nestin and βTubIII (Fig. 4C). When cultured in 3D-Matrigel, the cells formed clusters that co-expressed tdTomato and markers such as S100β (Fig. 4D). The cell clusters from the Matrigel cultures (Fig. 4E, left panel) were also fixed, embedded in paraffin and sectioned, before subjected to immunofluorescence. The results confirmed co-localization of tdTomato and βTubIII and GFAP (Fig. 4E, right panels). Together, these experiments suggested that at least part of the neuron-like cells derived from the Adiponectin-expressing adipose cells.

### Potential uses for brDFAT and wDFAT cells

**Figure 5:**
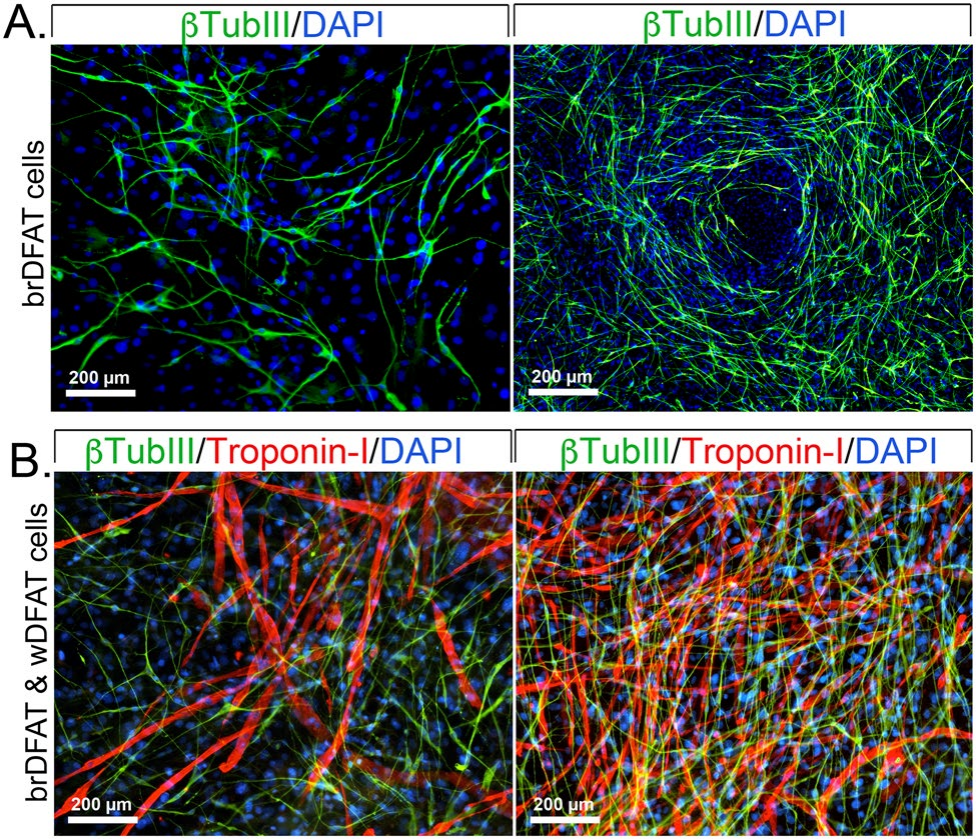
Potential use of mouse DFAT cells as a progenitor cell model. (**A**) Expression of βTubIII (green) in brDFAT cells after 10 days (left) and 15 days (right) in neural induction medium. (**B**) Immunolocalization of Troponin-I (red) and βTubIII (green) in cultures of combined wDFAT and brDFAT cells, which showed both cardiomyogenic and neurogenic differentiation after 10 days (left) and 15 days (right) in neural induction medium. DAPI (blue) was used to visualize nuclei.

In culture, the mouse brDFAT cells neurogenic cells frequently formed circular pattern after about 2 weeks, as shown in Fig. 5A by immunofluorescence for βTubIII. The pattern suggested activation of specific pathways for cell positioning that could be exploited to understand cell patterning. We then combined wDFAT cells, with demonstrated ability to undergo cardiomyogenic differentiation^16^, and brDFAT cells with neurogenic propensity. After approximately 14 days, several areas of combined cardiomyocyte-like and neuron-like cells were observed, with multiple connections between the two cell types (Fig. 5B). Thus, the cell types may be used for studies on how cardiomyogenic and neurogenic cells coordinate cell contacts during early differentiation.

### Human wDFAT and brDFAT cells show neurogenic propensity

**Figure 6:**
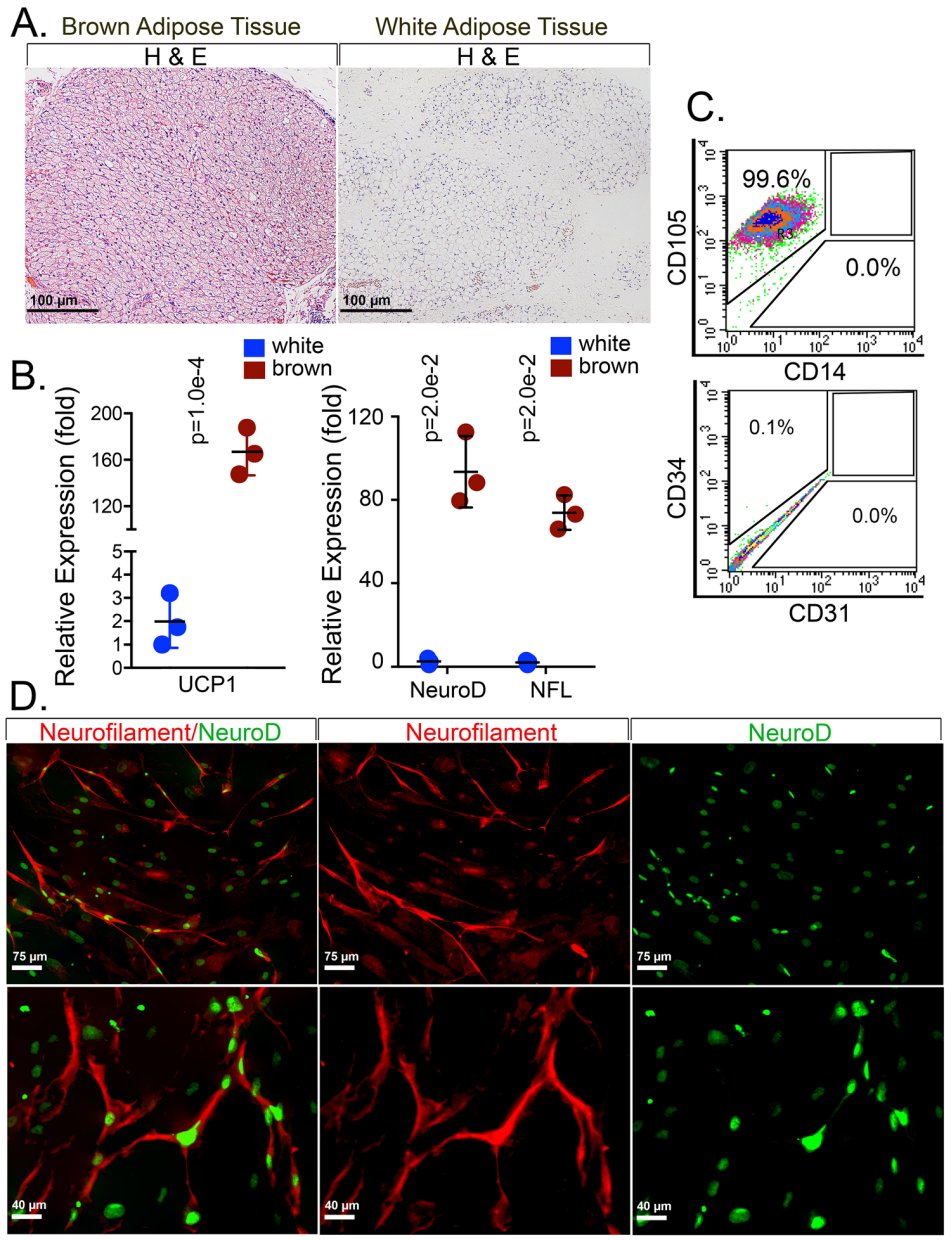
Neurogenic potential in human brDFAT cells. (**A**) H&E staining of human BAT (interscapular) and WAT (subcutaneous) from human autopsy specimens (from subjects less than 3 months of age). (**B**) Expression of Uncoupling protein 1 (UCP1), Neurogenic Differentiation 1 (NeuroD1) and Neurofilament (NFL) in white and brown adipose tissue from human autopsy specimen as determined by qPCR (n = 3 cell preparations). *P*-values for statistically significant differences are indicated (unpaired t-tests). (**C**) Expression of CD105 and CD34 in human brDFAT cells as determined by FACS on day 7 of culture. (**D**) Expression of NFL (red) and NeuroD (green) in human brDFAT cells after 15 days of culture in neural induction medium, as visualized by immunofluorescence. DAPI (blue) was used to visualize nuclei. Representative images from 3–5 experiments.

To determine if a similar ability to undergo neurogenic differentiation exists in human wDFAT and brDFAT cells, we prepared RNA and cells from human de-identified adipose tissue samples (Fig. 6A). RNA analysis by qPCR showed significantly increased expression of both UCP1 and the neurogenic markers NeuroD and neurofilament (NFL) in human BAT, as compared to WAT (Fig. 6B). The human brDFAT cells showed high expression of CD105 (99.6%) (Fig. 6C), which differed from the mouse brDFAT cells, where CD105 was only expressed in a small percentage of the cells. There was no detectable expression of CD14, CD34 or CD31. The human brDFAT cells were cultured in neurogenic medium for 14 days, and expression of the neurogenic markers NeuroD and NFL was examined by immunofluorescence. The results showed that cells with neurogenic morphology and expression of NeuroD and NFL appeared in the cultured brDFAT cells (Fig. 6D), supporting the existence of cell populations with ability to undergo neurogenic differentiation also in human BAT. Thus, precursor cells derived from both mouse and human BAT have the ability to undergo neurogenic differentiation and may serve as cell models for further studies.

## Discussion

Our results suggest an enrichment of neural precursor cells in mouse and human BAT as compared to WAT. This is consistent with the sympathetic nervous system being a key regulator of BAT thermogenesis and the enhanced abundance of sympathetic nerves in the BAT compared to the WAT^8^. Sympathetic innervation is also essential for cold-induced “beiging” of mouse WAT^17^. Cell populations from mouse and human BAT and WAT were able to undergo neurogenesis when the culture conditions were adjusted to stimulate neurogenic differentiation. At least part of these cells may be derived from Adiponectin-expressing cells as determined by cells expressing tdTomato under the control of the *Adipoq*-promoter. Neural precursor cells derived from the DFAT cells may be used as tools for studying neurogenic differentiation in adipose tissue, alone or in combination with other types of lineage differentiation.

Although the subpopulations derived from BAT and WAT showed capacity for neurogenic differentiation, the cells were not uniform in their differentiation. The expression of several neurogenic marker genes varied between the different subpopulation as determined by qPCR and immunofluorescence. In the mouse cells, the expression of GFAP and S100β, markers for early astroglial cell types and late astrocytes, respectively^18,19^, suggested differentiation along the astroglial axis. The expression of Olig2, however, suggested elements of oligodendroglial differentiation^20^. The neural stem cell marker βTubIII^21^ was fairly evenly expressed during the time course, potentially due to many cells remaining at a progenitor stage. Expression of NeuroD and NFL in the human cells supported the presence of precursor cells with capacity for neurogenesis^22,23^. Furthermore, the ability of the multipotent cells to undergo neurogenesis was dependent on the micro-environment in the culture dish, such as coating of the plates and addition of specific growth factors^24^. Finally, the method used to prepare wDFAT and brDFAT cells may promote certain cell populations depending on the lipid content and the buoyancy of the white and brown adipocytes, respectively.

Adiponectin-expressing cells appeared to at least in part be the origin of the cell populations with neurogenic capacity. The tdTomato Red co-localized with the neurogenic markers in both regular culture and 3D-Matrigel. However, it is not clear whether the neural precursor cells were derived from preadipocytes that expressed Adiponectin prior to lipid accumulation^25^ or if they were a results of dedifferentiation of lipid-filled adipocytes^26,27^.

Most adipose-derived stem cells that have been studied or used for regenerative purposes have been derived from WAT, which is generally available in large amounts from experimental animals and human subjects. Difficulties to obtain enough BAT from adult individuals is a clear obstacle. However, Silva et al.^28^ identified a clonogenic population of metabolically active stem cells from human adult BAT that could be expanded in vitro, and differentiate into metabolically active brown adipocytes as well as osteogenic, chondrogenic and white adipogenic lineages. Our study adds neurogenic differentiation to the potential use of BAT-derived stem cells. Because of the various lineage potentials of BAT- and WAT-derived cells, the cells also might be used to model interactions between different cell lineages such as such as neurogenic and cardiomyogenic cells. However, for systematic studies, it would be necessary to find a reproducible trigger to use in such experiments.

In conclusion, BAT-derived progenitor cells demonstrate neurogenic potential that can be modulated by culture conditions. When available, BAT might be a better source of neurogenic cells than WAT. To our knowledge, this is the first study to show that BAT-derived progenitor cells have neurogenic potential.

## References

[1] Feng N (2014). Generation of highly purified neural stem cells from human adipose-derived mesenchymal stem cells by Sox1 activation. Stem Cells Dev..

[2] Ohta Y (2008). Mature adipocyte-derived cells, dedifferentiated fat cells (DFAT), promoted functional recovery from spinal cord injury-induced motor dysfunction in rats. Cell Transplant..

[3] Saely CH, Geiger K, Drexel H (2012). Brown versus white adipose tissue: a mini-review. Gerontology.

[4] Berry DC, Stenesen D, Zeve D, Graff JM (2013). The developmental origins of adipose tissue. Development.

[5] Montanari T, Poscic N, Colitti M (2017). Factors involved in white-to-brown adipose tissue conversion and in thermogenesis: a review. Obes. Rev..

[6] Labbe SM (2015). Hypothalamic control of brown adipose tissue thermogenesis. Front. Syst. Neurosci..

[7] Bagchi M (2013). Vascular endothelial growth factor is important for brown adipose tissue development and maintenance. FASEB J..

[8] Bartness TJ, Vaughan CH, Song CK (2010). Sympathetic and sensory innervation of brown adipose tissue. Int. J. Obes. (Lond.).

[9] Jespersen NZ (2013). A classical brown adipose tissue mRNA signature partly overlaps with brite in the supraclavicular region of adult humans. Cell Metab..

[10] Sorensen I, Adams RH, Gossler A (2009). DLL1-mediated Notch activation regulates endothelial identity in mouse fetal arteries. Blood.

[11] Bagchi DP, MacDougald OA (2019). Identification and dissection of diverse mouse adipose depots. J. Vis. Exp..

[12] Jumabay M (2014). Pluripotent stem cells derived from mouse and human white mature adipocytes. Stem Cells Transl. Med..

[13] Jumabay M (2018). Combined effects of bone morphogenetic protein 10 and crossveinless-2 on cardiomyocyte differentiation in mouse adipocyte-derived stem cells. J. Cell. Physiol..

[14] Jumabay M (2012). Endothelial differentiation in multipotent cells derived from mouse and human white mature adipocytes. J. Mol. Cell. Cardiol..

[15] Yao Y, Zebboudj AF, Shao E, Perez M, Bostrom K (2006). Regulation of bone morphogenetic protein-4 by matrix GLA protein in vascular endothelial cells involves activin-like kinase receptor 1. J. Biol. Chem..

[16] Jumabay M, Zhang R, Yao Y, Goldhaber JI, Bostrom KI (2010). Spontaneously beating cardiomyocytes derived from white mature adipocytes. Cardiovasc. Res..

[17] Jiang H, Ding X, Cao Y, Wang H, Zeng W (2017). Dense intra-adipose sympathetic arborizations are essential for cold-induced beiging of mouse white adipose tissue. Cell Metab..

[18] Johnson K (2016). Gfap-positive radial glial cells are an essential progenitor population for later-born neurons and glia in the zebrafish spinal cord. Glia.

[19] Raponi E (2007). S100B expression defines a state in which GFAP-expressing cells lose their neural stem cell potential and acquire a more mature developmental stage. Glia.

[20] Ligon KL (2004). The oligodendroglial lineage marker OLIG2 is universally expressed in diffuse gliomas. J. Neuropathol. Exp. Neurol..

[21] Young A (2011). Ion channels and ionotropic receptors in human embryonic stem cell derived neural progenitors. Neuroscience.

[22] Seo S, Lim JW, Yellajoshyula D, Chang LW, Kroll KL (2007). Neurogenin and NeuroD direct transcriptional targets and their regulatory enhancers. EMBO J..

[23] Yuan A, Rao MV, Nixon RA (2012). Neurofilaments at a glance. J. Cell Sci..

[24] Gordon J, Amini S, White MK (2013). General overview of neuronal cell culture. Methods Mol. Biol..

[25] Hong KY (2015). Perilipin+ embryonic preadipocytes actively proliferate along growing vasculatures for adipose expansion. Development.

[26] Jumabay M, Bostrom KI (2015). Dedifferentiated fat cells: a cell source for regenerative medicine. World J. Stem Cells.

[27] Cote JA, Ostinelli G, Gauthier MF, Lacasse A, Tchernof A (2019). Focus on dedifferentiated adipocytes: characteristics, mechanisms, and possible applications. Cell Tissue Res..

[28] Silva FJ (2014). Metabolically active human brown adipose tissue derived stem cells. Stem Cells.

